# Study on “Atypical” Migraine Auras in the Pediatric Age: The Role of Cortical Spreading Depression and the Physiopathogenetic Hypothesis Arising from Our Clinical Cases

**DOI:** 10.3390/life12030450

**Published:** 2022-03-18

**Authors:** Vincenzo Raieli, Mariarita Capizzi, Antonio Marino, Giovanni Di Nardo, Umberto Raucci, Pasquale Parisi

**Affiliations:** 1 Child Neuropsychiatry Unit ISMEP, ARNAS Civico Palermo, Via dei Benedettini 1, P.O. Di Cristina, 90134 Palermo, Italy; 2 Child Neuropsychiatry School, University of Palermo, 90128 Palermo, Italy; mariaritacapizzi93@gmail.com (M.C.); antoniomarino94@tiscali.it (A.M.); 3 Department of Neuroscience, Mental Health & Sense Organs (NESMOS), Faculty of Medicine & Psychology, Sant’Andrea Hospital, 00189 Rome, Italy; giovanni.dinardo@uniroma1.it (G.D.N.); pasquale.parisi@uniroma1.it (P.P.); 4 Pediatric Emergency Department, Bambino Gesù Children’s Hospital, IRCCS, 00165 Rome, Italy; umberto.raucci@opbg.net

**Keywords:** aura, migraine, headache, cortical spreading depression, children, cortex

## Abstract

Migraine is a complex neurologic disorder by which several systems of the central nervous system (autonomous system, affective, cognitive, sensory and motor system) may be affected on different levels. About a fourth of migraine patients have migraine auras. The most common aura is the visual aura followed by the sensorial aura but motor deficits, as well as deficits of higher cortical centers (disorders of thinking, orientation, coherence, or concentration), may occur as well. In analogy with a headache diary, an aura diary can deliver important help in the diagnostic process of rare migraine manifestations and prevent the under-diagnosis of unusual migraine manifestations. Complex migraine manifestations are a diagnosis of exclusion, and a broad diagnostic work-up is necessary in order to exclude dangerous neurologic pathologies. In addition, here, we discuss the atypical clinical presentation and possible physio-pathogenetic related aspects of these atypical migraine aura features in the developmental age. In addition, we wanted to stress and analyze the clinical aspects of our children/adolescents with atypical auras, which seem to be more difficult to frame with the mechanisms originally proposed to explain the physio-pathogenetic relationship between CSD and aura. Finally, we discuss in detail the complex aspects of this topic on the basis of available data and propose new terminology: “*Multiple, Synchronous and Asynchronous, Cortical and Subcortical Spreading Depression*”.

## 1. Introduction

Migraine is an extremely complex disorder in its pathophysiological mechanisms, and a critical analysis of the literature and the available clinical data suggest that the various theories may appear simplistic, frequently underlining only one aspect of the disorder [1]. Most of the studies on a clinical case series or on a small group of migraineurs studied with functional neuroimaging or neurophysiological methods have revealed many common aspects in patients diagnosed as migraineurs [2]. However, the use of syndromic diagnostic criteria (association of signs and/or symptoms) can define many subjects as migraine sufferers who are very different from each other in the phenotype of the attack, in its chronology, in its evolution, in the response to treatments, etc., therefore, a broad diagnostic work-up is necessary. The current headache classification [3], taking note of a frequent variability of the aura symptoms, admits that subjects suffering from migraine can report a series of characteristics associated with each other, freely, and with wide variability in the temporal sequence, onset, and duration of the signs and symptoms of the aura; however, in addition to ignoring the possible presence of cognitive dysfunctions of cortical origin (now well documented in the migraine aura) [4]. A distinctive feature of the ictal semiology of the migraine aura is the gradual onset and progression of semiology, in contrast to the sudden onset, typical in epilepsy, ischemia, or hemorrhage [1,2]. Additionally, unlike ischemia, positive, visual, and sensory-like symptoms (e.g., flashing lights and paraesthesia) are more common than negative symptoms in the migraine aura. These symptoms would reflect, in fact, an underlying physiological phenomenon that begins and spreads slowly (CSD). Visual aura symptoms are by far the most common and occur in 90% or more of patients, followed by sensory, motor, and speech symptoms, with visual symptoms often presenting as only manifestations [5,6,7]. The reason for this predilection of the migraine aura mainly involving the visual cortex remains unclear, and the onset of isolated non-visual semiology is unusual. Nonetheless, the visual semiology can be followed progressively by the other symptoms [1,2,3]. On the need to integrate the more defined pathogenetic aspects of the main clinical feature of migraine with very discordant clinical and instrumental data in individual patients, an important example comes from the study of the “aura”, one of the most typical symptoms of migraine manifestation that in the last thirty years has found the theory of “Cortical Spreading Depression” (CSD) as the main and perhaps the only plausible explanation of the pathophysiological mechanism [1]. Already, a few years ago, several studies were trying to go beyond the theory of CSD as classically reported, trying to explain the visual aura through a computational model that would also account for the different morphologies of the visual aura and suggesting a thorough anamnestic description of the other forms of aura to extend this computational model [8], or to explain certain aspects of the migraine aura (see amnesia, cognitive disorders) with the spread of CSD, not only in the cortical but in subcortical structures via connecting cortical structures, such as the entorhinal cortex [9] or, finally, on the basis of accurate clinical descriptions, they revealed the possibility of the presence of selective cortical cognitive disorders and suggested the possible simultaneous activation of different cortical areas by separate CSDs [10].

However, in recent years (2015–2022), even from researchers who have conducted significant studies in support of this theory, numerous contradictions have begun to highlight its inadequacy and plausibility and to suggest the need to clarify and modify some aspects [11,12,13,14,15,16], and also in the light of data that come from clinical practice [6,7,17]. In fact, in light of both experimental and clinical data, the theory of CSD was subjected to revision and criticism for its poor adaptation to the clinical presentation of the migraine aura, at least in a relevant percentage of “presentations with atypical clinical pictures” [2,11,12,13,14,15,16]. 

To underline that this discrepancy can be particularly accentuated in the migraine auras observed in the developmental age, here, we describe and discuss some pediatric clinical cases extracted from our outpatient cases series. We present a series of pediatric subjects suffering from migraine with aura who show atypical characteristics that cannot be explained according to the general commonly accepted theory and to discuss the critical points of the general theory of “Cortical Spreading Depression” in the light of both our “atypical aura cases” and other scientific data coming from the most recently published literature. 

We also propose a physio-pathological point of view to explain with a “common physio-pathological hypothesis” the relationship between CSD, trigemino-vascular system (TVS) activation, cephalalgic phase (headache), and “aura semiological manifestations”.

## 2. Materials and Methods

We retrospectively reviewed all the outpatient and inpatient records of children (aged between 8 and 17 years) admitted to our headache centers in Palermo (Sicily, Italy), from 1 April 2010 to 31 March 2020, diagnosed with migraine, coded according to ICHD-3 IHS criteria [3]: 114 migraine with aura (M/F), equal to 14% of the entire sample. From this population of migraine sufferers with aura, we selected 16 cases whose semiological-temporal picture was difficult to contain within the limits accepted by the international classification on headaches, according to the etio-physiopathogenetic assumptions suggested by the theory of “Spreading Depression (CSD)”, as it is currently enunciated. These patients with “atypical auras” were selected starting from the original case series of the Pediatric Headache Centers in Palermo, during 3 ad hoc meetings, which were initially attended by all the authors of the manuscript. However, the final selection of the 16 patients to be included for atypical auras was taken jointly, with a final discussion by VR and PP. Following this final meeting, we decided to divide our 16 children with “atypical aura” into five different types:

Type 1. If the diffusion wave does not satisfy the posterior–anterior spreading progression modality according to the classically described physiopathology for CSD;

Type 2. If the chronological sequence in the succession of symptoms does not follow the temporal progression, typical of CSD, and the cortical homunculus is not respected;

Type 3. If the time intervals of onset between symptoms are not justified by the current “(CSD)” theory;

Type 4. If there is an association of clinical symptoms that are peculiar or difficult to justify in light of the CSD theory;

Type 5. If there is a correlation of the aura with pain that cannot be justified by the pathophysiology of CSD, both for time course and for the area involved in the pain.

All patients underwent a thorough diagnostic procedure including: Brain MRI, inter-ictal-awake/sleep EEG and (where possible) an ictal-awake-EEG (3/16 subjects), Doppler ultrasound of the supra-aortic arterial cranial vessels, cardiac audit and cardiac Doppler ultrasound, blood tests to evaluate liver, kidney and blood coagulation functions.

Written informed consent was obtained from the patients’ parents to publish this paper.

## 3. Results

According to the criteria (five types) as cited above, we grouped our 16 children with atypical auras as followed, specified in detail (see Table 1): 

Type 1: case numbers 8, 10, 11, 13, 15, 16.

Type 2: case numbers 4, 10, 13, 9, 14,16.

Type 3: case numbers 1, 3, 5, 12, 14, 16.

Type 4: case numbers 2, 6, 7, 11, 13, 15.

Type 5: case numbers 1, 4.

## 4. Discussion

The migraine aura is defined by the 3rd classification of the IHS (ICHD-3 [3]) as recurrent attacks, lasting minutes, of unilateral reversible visual, sensory, or other symptoms of the central nervous system, usually developing gradually and followed by headache and associated migraine symptoms. The IHS classification [3], taking note of a certain variability of symptoms, admits that migraine sufferers can complain of symptoms freely associated with each other and not always complete (at least three characteristics of criterion C must be present), does not seem to realize that some of these aspects can invoke mechanisms different from each other, which makes it difficult to have a single interpretative pathogenetic theory (how the relationship between CSD and aura onset is currently defined). A theoretical model of migraine aura must take into account the typical gradual onset and progression, in contrast to the sudden onset typical of epilepsy, ischemia, or hemorrhage. Additionally, unlike ischemia, positive, visual, and sensory-type symptoms (e.g., flashing lights, paresthesias) are more common than negative ones. These symptoms would reflect an underlying physiological phenomenon starting slowly and spreading slowly. The clinical observations that 90% or more of patients show visual symptoms followed by sensory, motor, and speech symptoms, suggest that the occipital cortex is mainly involved in the start of migraine aura [5,6,7]. Based on these clinical observations, the Leao study [17] suggested the correlation between the migraine aura and the experimentally observed Cortical Spreading Depression (CSD) as a pathophysiological mechanism. Given these similarities and the experimental data produced, the CDS has become the most accredited, perhaps even the only hypothesis, to explain the migraine aura over the last thirty years.

The CSD was summarily described by Leao [17] as the suppression of spontaneous EEG cortical activity evoked by electrical/mechanical stimulation of the brain, that spreads across the cortex, characterized by an initial short-lasting depolarizing wave following from the prolonged depression. This wavefront spreads across the cortex along all directions for about 3 mm/min, similar to the calculated aura speed by Lashley [18]. Successively, several studies [1] showed that CSD is the expression of neuronal and glial cortical depolarization, followed by sustained hyperpolarization. The CSD effects provoke changes in the cortical microenvironment varying the intra/extracellular ionic concentrations (see calcium, potassium, hydrogen, and sodium), and the release of serotonin, nitric oxide (NO), and glutamate. In humans, in vivo, the CSD was observed indirectly by the perfusion studies that underlined the metabolic/electrocortical changes and showed the alterations of cortical blood flow with initial hyperemia followed by sustained oligoemia. Past studies have experimentally shown that CSD was the possible link between the activation of the trigemino-vascular cascade and the triggering of migraine pain (headache). Accordingly, the hypothesis of silent CSD (involving clinical mute cortical areas) was suggested to explain the headache in subjects with migraine without aura [19].

Recently, Bolay et al. [14] underline some aspects that are difficult to interpret, such as, for example, a CSD that starts from a gyrus or sulcus of the cerebral cortex, causes a visual aura and, within 20 min, causes paresthesias, with the involvement of the somatosensory cortical areas, or even when these two phenomena arise simultaneously at the cortical level, as observed in our patients and in other clinical series [10]. Yet, CSD is usually limited to a few gyri in the primate brain, however, for multiple aura symptoms to occur sequentially, they propagate over numerous sulci along the human cerebral cortex [14]. They conclude that “*the simultaneous occurrence of different auras in different patients leads to the deduction of a different mechanism from a “simple” progressive wave of depolarization that crosses the cerebral cortex, and that it could be important to account the thalamus as a hub that provides extensive connections among the visual, somatosensory, language and motor cortical areas…*” [14]. Other authors underline the possible role of other cortical structures (see entorhinal cortex) to connect primary cortical areas with subcortical structures by CSD [9].

In addition, Dahlem et al. [20] hypothesize the presence of CDS patterns localized and influenced by cerebral cytoarchitectonics, which are variable from patient to patient (underlining the importance of individual variability), and also suggesting the existence of a general inhibitory signal that is confused with CSD in studies of cortical blood flow. Furthermore, they observe that a CSD that spreads radially with the calculated speed of 3 mm/min, without taking into account the vascularization and cytoarchitectonics, is not compatible with a sequence of visual-sensitive-aphasic aura lasting 180 min total (according to the IHS criteria) [20]. This suggests that the pathogenetic mechanism of the aura cannot be interpreted in the current modalities described for CSD and needs to be modified, or, in any case, individualized for each patient.

Finally, a more recent synthetic point of view, which summarizes the current evidence that modifies the interpretative model of CSD (adapted to the migraine aura), was proposed by Hadjkani and Vincent [13]. They suggested that a migraine is more than a chain of events linked together in a genetically susceptible brain and that it can be interpreted as a series of networks or components activated in different sequences and loci, with consequent localization of pain and different aura, and with variable temporal relationships, different possible associations with other symptoms, and with differences both infra and intraindividual. It was also underlined that in the CSD induced in the gyrencephalic feline cortex, secondary CSD events spread in parallel to the gyrus where the first CSD starts originally, encompassing a significantly smaller cortical area with a significantly slower speed [13]. These authors claimed that has not still supported the fact that secondary or parallel CSD waves could induce a prolonged aura in migraineurs. Furthermore, the occurrence of aura symptoms in sequence with delayed time intervals between them or simultaneously suggest the occurrence of multiple CSD and that these multiple CSD waves may arise in different points of topography and time [13].

Charles [2] also expressed his point of view and some perplexity about the way CSD spreads in the migraine aura, suggesting that *… “If the pathophysiologic mechanism of the migraine aura is indeed traveling in a more linear fashion along a gyrus or sulcus, this raises multiple other interesting issues. First, it is not clear by what path it could travel from the occipital cortex to the sensory cortex to the motor cortex. Second, the blood flow changes that have been observed in migraine may be much more extensive than changes in brain parenchymal activity that are responsible for aura symptoms. Finally, if migraine aura mechanisms do indeed contribute to pain, then it is clear that the distribution of headache is not correlated with the spatially limited location of the changes in brain activity that cause aura.”*

If the CSD model presents several criticalities in explaining the migraine aura (while remaining the best model to explain the aura triggered by the involvement of cortical areas, primarily visual, of which, Dahlem and Chronicle further elucidated the mechanisms of the “visual aura” by means of a computational model capable of predicting variations in visual disturbances based on the organization of the visual cortex [8]), it is even more difficult to explain the rare complications of the aura migraine, such as retinal migraine and migraine status, or even explain the pathophysiology of the brain-stem aura [14]. In accordance with our point of view, recently other authors [14,21,22] have suggested that there is no easy explanation for the sequence of visual aura followed by sensory aura in opposite body sides or focal sensory symptoms in one region (lips, mouth).

The above, in our opinion, underlines the need to modify or rather expand the pathogenetic model based on an accurate clinical description of the patients, especially those with atypical aspects, in order to suggest possible different modalities that can also “incorporate” and “coexist” with the CSD hypothesis. Here, we present and discuss some clinical cases that highlight particular characteristics of the migraine aura that may underlie and suggest peculiar additional pathophysiological mechanisms.

At this point, it is essential to summarize the main points (regarding the correlation between the neurophysiological aspects in CSD and the clinical ones of the “migraine aura”) that we must expect in light of the anatomic–physiological principles linked to the theory of CSD, comparing these theoretical concepts (commonly accepted by the scientific community) with the “atypical characteristics of the aura” in our children, here described in Table 1. We want to point out the discrepancy between the aura picture in our children, whose clinical picture (type of signs/symptoms and temporal sequence) is difficult to explain with the CSD theory. The main considerations peculiar to our case series and the discrepancies with the “pathophysiological rules” expected in accordance with the CSD theory are listed below:

- Considering the progressive and radial diffusion of CSD, while taking into account that diffusion can be hindered by gyration, in many cases, it can be expected that it will spread from the area of appearance, initially to the nearest and subsequently possibly slower, involving the progressive modification of symptoms, sequentially (for example, simple visuals—complex visuals—associative areas). On the other hand, our cases (see Table 1: 1, 8, 10, 11, 13, 16) cannot be easily explained according to this point of view.
- The duration of positive symptoms and the expression of a depolarizing wave (if present) must be much shorter and necessarily precede the negative symptoms that follow as a consequence of the more prolonged wave of hyperpolarization (depression of electrical activity). In this respect, if we look at case numbers five and nine (see Table 1), we cannot note positive symptoms preceding or associated with any visual negative ones.
- If the CSD wavefront spreads across the cortex along all directions for about 3 mm/min, we should expect that progressively, from the starting point, the various neighboring cortical areas are affected, radially, in every direction. Conversely, our cases (1, 2, 4, 11, 16) do not show such diffusion but show jumps from one cortical region to another, even simultaneously (as foci that ignite in a scattered, distant and synchronous way), or (see case 15), the aura manifests itself extremely localized at the level of sensitive/sensorial areas, or shows a retrograde progression from the sensitive areas towards the occipital cortex, but not (as one would expect) at the same time towards neighboring areas in every direction (see cases 8, 11).
- The individual patient should show certain stability of the cerebral starting point of the aura (e.g., occipital cortex) at least during the same episode. Instead, in our clinical case number 3, the semiological starting point is occipital but, once the aura has disappeared, only a sensitive aura reappears during the same attack.
- The cortical refractory period should prevent the reappearance of positive/negative symptoms in the affected cortical area, within a set period of time (refractory period), which can have a different length according to different areas. Conversely, our clinical case number 8 presents a “sensitive march”, then follows a visual aura and, subsequently, presents dysarthria; where, in reality, we should find mute cortical areas (due to the state of refractoriness), or, albeit in a retrograde sense, we should have witnessed the appearance of semiological manifestations in a different order, with a sensory–linguistic–visual sequence. Moreover, the duration of the complex aura predicts a normal cortical activity where the aura first appeared following progressively the other interested cortical areas. Moreover, if the aura lasts beyond a certain time, the diffusion of positive/negative symptoms restarts or persists unchanged for the prolonged duration of the aura.
- In clinical cases 1, 4, and 11, the unilateral pain should be localized contralateral to the aura. In fact, the spreading depression theory suggests that the CSD is able to activate the trigeminal fibers in the homolateral dura mater layer of the homolateral hemisphere; however, in our three cases it happens vice versa, and the spreading pathway of the pain to the contralateral hemisphere is not clear (case 1).

Thus, taking into account the above-stressed reflections, here we suggest, in particular, the possibility that local cortical focuses (and even subcortical focuses, with different activation thresholds, which vary over time and can also be related to the stage of development) of “Spreading Depression”, can have an origin, both simultaneously and in succession, and consequently the aura that occurs does not depend on the progressive irradiation of the CSD (radial diffusion of the CSD, in all directions, starting from the cortical point of origin) which would not, by itself, as formulated till now, be able to explain these clinical pictures of “atypical auras” (e.g., see cases 1, 2, 10, 11 in Table 1 and other clinical series [10]). Our hypothesis could be defined as “Multiple, Synchronous and Asynchronous, Cortical and Subcortical Spreading Depression”. Our hypothesis could also be supported by other recent experimental studies that have demonstrated the possibility of cortical–subcortical onset of local areas of “spreading depression” and even a possible role of the thalamus was also demonstrated in the cortical–subcortical activation networks [23] or, as Vinogradova [24] has shown, that the development of intense seizures in the cortex leads to the initiation of spreading depression in multiple cortical–subcortical sites of both hemispheres.

This activation of subcortical structures by CSD may also be transmitted from the primary cortex to subcortical structures via connecting cortical areas (see cortex entorhinal [9]).

## 5. Limitations of the Study

Our study has some limitations: (1) The limited number of patients with atypical auras compared to the number of subjects with typical migraine auras; however, it should be emphasized that the main reason for this article is to highlight how the theory of CSD, as classically expressed currently, it is not able to fully explain the frequent clinical data reported by us and other authors [6,7], so it is urgent to modify it, or to assume that there are different pathophysiological mechanisms underlying the onset of a “migraine aura”; (2) The clinical description, particularly from children and adolescents, depends on the ability to remember the details of the aura precisely, although it should be remembered that in almost all patients, their story was collected within a few hours of the onset of the aura or immediately after the admission to the pediatric emergency department, and in-depth anamnestic with subsequent hospitalization; (3) The lack of functional neuroimaging data in the ictal phase does not allow us to accurately support our possible alternative pathophysiological hypotheses that we put forward here. On the other hand, both the unpredictability of the episodes and their non-particular frequency, often present in the pediatric age, as well as the ethical aspects, make it very difficult to be able to acquire these data, particularly in the developmental age.

## 6. Conclusions

In conclusion, the auras of our patients do not have an easy explanation in relation to what is predicted by the classic theory of CSD, suggesting modifications of the same or the possibility that different mechanisms coexist that can be activated in some patients. On the other hand, the same diagnostic IHS criteria for the migraine aura, due to the possibility of different combinations not compelling for the mandatory satisfaction of all the criteria (see march, duration, start, etc.), implicitly imply the heterogeneity of the migraine aura, which probably underlies various physiopathological mechanisms that can prevail or co-occur with each other.

Awareness of these complex clinical aspects must induce clinicians to collect an accurate description of the migraine aura, which often in children and adolescents can be extremely characteristic and suggest a possible pathophysiological hypothesis to be investigated. Furthermore, the presence of “atypical” aspects does not necessarily suggest the presence of possible secondary causes of the migraine attack, therefore, in the presence of aspects such as the relationship with the typical migraine pain, the recurrence of episodes, the interval of full well-being, the familiarity of migraine due to aura can help in undertaking a non-excessive, non-invasive diagnostic process. 

Regarding alternative pathophysiological explanations of the manifestations of our sample we can suggest that:

(1) The “Spreading Depression” is not an exclusively cortical phenomenon. It can originate in a synchronous and asynchronous mode in different points of the brain (at the cortical–subcortical level);

(2) The activation of the Trigeminal-Vascular System (TVS) (which is at the origin of the “headache phase”) is not an exclusive prerogative of CSD because the TVS system can be activated at the cortical–subcortical level independently of a wave of “Spreading depression”.

(3) At the cortical level, an “epileptic discharge”, the “cortical spreading depression”, the activation of TVS, and the “headache phase” can sometimes completely overlap in the “Ictal Epileptic Headache” [25,26,27].

(4) The complexity of the networks involved in the etiopathogenesis of the headache and migraine aura is further complicated in its phenotypic expression in the developmental age by the development of the central and peripheral networks of the nervous system and the related processes of maturation and myelination in continuous evolution [28].

(5) Finally, in our opinion, our suggestions and reflections arising from our reported clinical cases with atypical auras expand the CSD theory with a modification that does not cancel but rather includes with a “greater and broader” etiopathogenetic complexity the original hypothesis of CSD. In particular, our point of view still keeps the hypothesis alive in its original interpretation, only widening the boundaries of its action, topography, and the role and interactions at the various levels (cortical and subcortical) in the stations along the trigemino-vascular activation (TVS) axis, redefining the theory as “**Multiple, Synchronous and Asynchronous, Cortical and Subcortical Spreading Depression network**”.

## Figures and Tables

**Table 1:** Case series of atypical aura.

	Name, Sex, Age	Date of Visit	N. Episodes	Type of Aura	Aura Sequence	Duration	Particular Description of Episodes
1	M, 13.2 ys	5 November 2020	2	V-S	V-S	20 visual; <15 sensitive	Before appearance of left hemianopsia, subsequent appearance of headache on the left side which then moves to the right and then becomes bilateral. During headache, visual aura already disappeared for some time (at least 30 min), the appearance of a marked sensation of falling asleep to tongue and lower lip, without other irradiation (perhaps sensation of confusion). Previous aura.
2	F, 16.5 ys	12 May 2020	3	S-S-C-L	V-S-C-L	1 h	Amaurosis left followed by paraesthesia in hemilip, sx and successive difficulty reading (language disorder) and formulating thoughts (cognitive disorder).
3	F, 17 ys	15 February 2017	>5	V-V-S	V-S (first appearance) V-S-L (second appearance)	20 m. × aura mode.	Hemianopsia dx and successive headache appearance, after disappearance visual aura sensitive aura appeared (march: hand–arm–face), after 2 h disappearance sensitive aura, in presence of persistent headache the sensitive aura re-appeared after temporal interval of about two hours (two steps of aura in the same migrainous attack).
4	M, 12.6 ys	28 May 2020	3	S-Mo	S-L-Mo	90 min	Appearance of falling asleep and swelling of the tongue with labial rhyme deviation due to dx deficit—follows dysarthria—deficit dx hand and wrist and paresthesia of dx lower limb—follows headache on the right side.
5	F, 13.2ys	7 April 2000	1	V	-	>4 h	Left amaurosis without positive symptoms lasting a few days and onset of right fronto-temporal headache.
6	M, 11.6 ys	28 April 2017	1	V	V-vertigo	>3 h	Right hemianopsia at central start as triangle with black scotoma and white phosphorescent sides, disappeared from the center.
7	M, 12.7 ys	13 February 2017	1	C	-	20 min	Upon awakening headache with difficulty in reading and understanding of the writing.
8	F, 15.1 ys	21 June 2013	2	V, C-L	S-V-L	<1 h	One episode starts with sensitive symptoms with a typical right gear followed by visual disturbances (phosphenes and spectrum in the right hemicampus) followed by the appearance of language disorder, particularly in production. A further isolated episode only visual.
9	F, 12.10ys	17 September 2016	>4	V, S	V-S	<20 min	Sudden complete blurring of short-term vision followed by falling asleep of the right hand, without progression lasting less than 20 min followed by violent frontal headache with 2 episodes of vomiting lasting hours.
10	M, 10 ys	9 June 2015	2	S-Mo-V	S-Mo-V	>1 h	An episode with paresthesia to the left thigh, left foot, hand, arm, left half-face, headache, and appearance of visual aura (bright colored stripes). Other motor-sensitive analogous episodes followed by dysarthria, no visual disturbances.
11	M, 13.10 ys	6 May 2017	2	C, L, V	C-L-V	>1 h.	Appearance of estrangement sensation associated with speech disorders with phonological errors and appearance of sx hemianopsia. It follows a sx and intense left temporal headache. Analogous episode 48 h later, during hospitalization.
12	F, 9 ys	7 December 2010	4	S, D, V	S-D, V		Gradual, pulsating one-sided left headache, during which paresthesias to the right hemitongue appeared and dysarthria, followed by paresthesias in the hand with irradiation to the elbow, followed by remission within 20–30 min. After about thirty minutes from the disappearance of neurological symptoms, while the headache persisted, some paresthesias reappeared in the right hand extending to the elbow and subsequent extension to the tongue with deviation of the right lip rhyme. The aura would have lasted about 30 min. New episode about 15 months later with the previous characteristics of a sensitive type with the exception of shorter duration and without presenting the onset “two-stroke”, the EEG track, executed in the presence of headache, highlighted a marked asymmetry of the background rhythm, slowed to the left. After 6 months, an episode of visual aura that, from the center, gradually widened towards the periphery bilaterally with a front characterized by colored stripes without that there was a complete obscuration of the visual field.
13	F, 12 ys	18 May 2010	4	H, S	U-S		Episode characterized by the appearance of sounds and voices “as heard far away”, located mainly in the left ear, then paresthesias to the tongue and shortly after to the left hand up to the wrist. The duration of the aura was about 10–20 min. At the end of the episode, frontal headache followed almost immediately, pulsating and discreet intensity.
14	F, 11.7 ys	4 April 2019	>6	S	S	<5 min	Numerous ongoing episodes of headache prevalent on the left, appearance of paresthesias in right hand and quick feeling of swollen tongue, no visual disturbances.
15	F, 14 ys	15 February 2021	>6	S	S	>20 min	Localized paresthesias to the lips with swelling of the same.
16	M, 13.7 ys	5 July 2021	>6	V, S-L	S-M-L	5–15 min	At 1st aura episode, contemporary appearance of paresthesias III-IV-V fingers on the right hand and I and II right toes with homolateral weakness, after 15 min severe bitemporal headache and aura ended; 1 h after dysarthria and static buccal rhyme deviation to the right of time 5–10 min. He repeated episode after about 15 min and then after about 4 h. The headache lasted throughout the day. History of severe weekly headache and some episodes associated with visual aura.

C: cognitive disorders; D: dysarthria; F: female; H: hearing disorders; L: language disorders; M: male; Mo: motor disorders; S: sensitive disorders; V: visual disorders; ys: years.

## Data Availability

The data was contained in the clinical standard files.

## Permissions

We do not need any permission for images or tables or text. The authors have received a patient consent form from any patient, and they have it in case they are requested by the editor.

## Abbreviations

CSD	Cortical Spreading Depression
ICHD-3	International Headache Classification
TVS	Trigemino-vascular system
